# Next‐generation sequencing of baseline genetic mutations and outcomes of eltrombopag and azacitidine therapy in patients with myelodysplastic syndromes and thrombocytopenia: Data from the SUPPORT clinical trial

**DOI:** 10.1002/jha2.694

**Published:** 2023-05-22

**Authors:** Pedro Marques Ramos, Jeea Choi, Catarina D. Campbell, Ying A. Wang, Celine Pallaud, Michael Dickinson, Amit Verma, Moshe Mittelman, Uwe Platzbecker, Honar Cherif, Pierre Fenaux

**Affiliations:** ^1^ Novartis Pharma AG Basel Switzerland; ^2^ Novartis Pharmaceuticals Corporation East Hanover New Jersey USA; ^3^ Novartis Institutes of BioMedical Research Cambridge Massachusetts USA; ^4^ Novartis Global Drug Development Cambridge Massachusetts USA; ^5^ Present address: Bayer Pharmaceuticals Corporation Cambridge MA USA; ^6^ The Sir Peter MacCallum Department of Oncology The University of Melbourne Melbourne Australia; ^7^ Division of Medical Oncology Department of Medicine Albert Einstein College of Medicine New York New York USA; ^8^ Tel Aviv Sourasky Medical Center Tel Aviv University Tel Aviv Israel; ^9^ Medical Clinical and Policlinic Hematology and Cellular Therapy University Hospital Leipzig Leipzig Germany; ^10^ Department of Medical Sciences Uppsala University Uppsala Sweden; ^11^ Hôpital Avicenne Assistance Publique‐Hôpitaux de Paris/University Paris XIII Bobigny France

**Keywords:** azacitidine, myelodysplastic syndromes, NGS, thrombocytopenia, thrombopoietin receptor agonist

## Abstract

Eltrombopag has been previously shown to be effective in reversing azacitidine‐mediated thrombocytopenia. This was further investigated in the SUPPORT trial, a phase III study assessing the efficacy/safety of eltrombopag plus azacitidine in patients with intermediate‐ to high‐risk myelodysplastic syndromes and thrombocytopenia. The results did not support a clinical benefit for the addition of eltrombopag to azacitidine. We investigated if the somatic mutational profiles in the patient cohort were associated with treatment outcomes. Based on the available data, we observed no imbalance in the mutational profiles between treatment arms or a clear association between identified somatic mutations and clinical outcomes.

1

Thrombocytopenia occurs in 40%–65% of patients with myelodysplastic syndromes (MDS) and is associated with poor prognosis [1]. Hypomethylating agents used to treat advanced MDS, such as azacitidine, are associated with the development or exacerbation of thrombocytopenia [2]. It was previously shown in phase I and II trials that eltrombopag can reverse thrombocytopenia caused by azacitidine [3, 4].

The phase III SUPPORT trial (NCT02158936) investigated the efficacy and safety of eltrombopag plus azacitidine compared with placebo plus azacitidine in patients with intermediate‐ to high‐risk MDS and thrombocytopenia. The primary endpoint of platelet transfusion independence in cycles 1–4 of azacitidine therapy was inferior in the eltrombopag versus placebo arm (16%, *n* = 28/179 vs. 31%, *n* = 55/177, respectively), and the proportion of patients with progression to acute myeloid leukaemia (AML) tended to be higher in the eltrombopag than in the placebo arm (15%, *n* = 27/179 vs. 9%, *n* = 16/177, respectively) [5]. This report focusses on whether genetic factors contributed to the findings.

Recently, somatic mutations in >40 genes have been identified in the molecular profile of MDS, and distinct molecular subgroups with different prognoses or risks of progression to AML have emerged [6]. Driver mutations have been found in a variety of genes involved in RNA splicing (e.g., *SF3B1*), DNA methylation (e.g., *TET2*), chromatin modification (e.g., *ASXL1*), transcription regulation (e.g., *RUNX1*), DNA repair (e.g., *TP53*), signal transduction (e.g., *NRAS*) and the cohesin complex (e.g., *STAG2*) [7].

Point mutations in *RUNX1*, *TP53* and *NRAS* were associated with severe thrombocytopenia and an increased proportion of bone marrow blasts, whereas point mutations in *TP53*, *EZH2*, *ETV6*, *RUNX1* and *ASXL1* were associated with shorter overall survival [8]. To address the possibility of genetic factors contributing to the outcome of SUPPORT, we performed biomarker analyses by next‐generation sequencing (NGS) on 53 genes previously reported to be commonly mutated in MDS, including a set of 18 genes associated with poor prognosis (Table SI) [5]. We analysed the mutational profiles of individuals within SUPPORT to investigate whether (1) specific genetic mutations could be associated with the rate of progression to AML in each of the treatment arms; (2) an imbalance in genetic mutations might explain the increased progression to AML in the eltrombopag arm; or (3) treatment may have influenced patterns of clonal evolution.

Out of a total of 356 patients randomised to either eltrombopag or placebo, both in combination with azacitidine (intent‐to‐treat population; Figure S1), peripheral blood samples were taken at baseline in 211 patients (NGS population; eltrombopag, *n* = 101; placebo, *n* = 110). Both arms in the NGS population showed similar demographic and disease characteristics (Table S2), with no apparent differences in patient characteristics between the NGS (*n* = 211) and intent‐to‐treat (*n* = 356) populations. Further description of the materials and methods used is included in the Supporting Information (Materials and Methods).

The proportion of patients with mutations in ≥1 of the 53 MDS‐related genes at baseline was numerically lower in the eltrombopag arm than in the placebo arm (85.1% vs. 91.8%; odds ratio [OR] 0.51; 95% confidence interval [CI] 0.19–1.32). Similarly, the proportion of patients with mutations in ≥1 of the 18 prognostic genes was lower in the eltrombopag arm (76.2% vs. 87.3%; OR 0.47; 95% CI 0.21–1.02).

A previous study reported an association between patient outcome and the number of oncogenic mutations [9]. Therefore, we investigated the relative distribution of patients in the two arms by the number of mutations and observed similar frequency in both arms for all groups (Table S3).

Subsequent analyses focussed on a set of 11 genes for which mutations were found in >5% at baseline in at least one arm. The analyses showed no evident differences between the arms in the proportion of patients with mutations, although baseline frequencies of mutations in *TET2*, *TP53* and *DNMT3A* were numerically (5%–6%) higher in the placebo versus the eltrombopag arm (Figure 1A,B). No differences in the allelic frequencies of mutations in any genes or the number of variants of each gene were evident (Figure S2).

**FIGURE 1:**
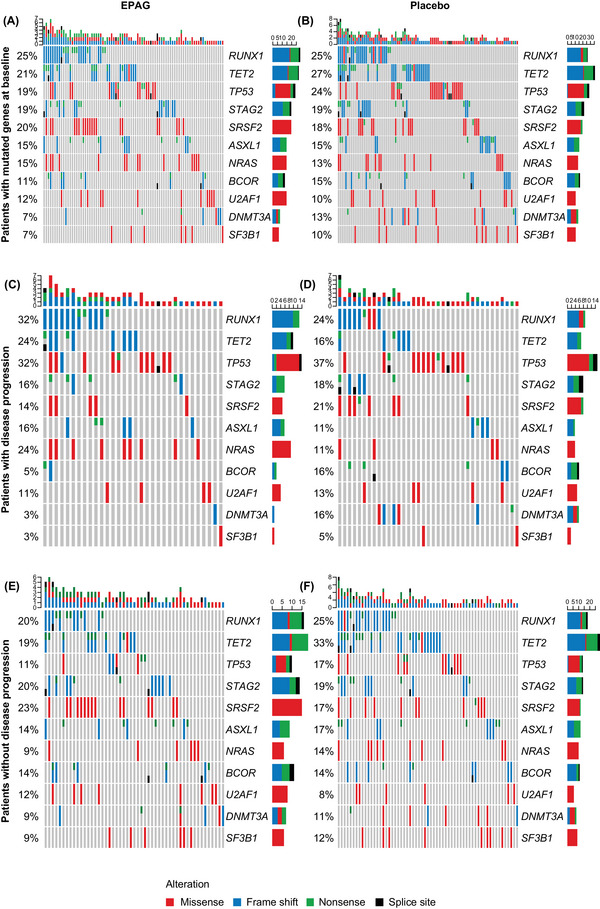
Baseline mutations according to treatment arm in the 11 most commonly mutated genes at baseline in patients with myelodysplastic syndromes (MDS) and baseline frequency of gene mutations among patients with or without disease progression to acute myeloid leukaemia (AML). (A and B) OncoPrints of gene mutations in MDS‐related and prognostic genes are shown by treatment arm for the 11 most commonly mutated genes. Percentages represent the percentage of patients in each arm with a mutation in the indicated gene. (C and D) Genomic alterations in patients with disease progression, by treatment arm. (E and F) Genomic alterations in patients without disease progression, by treatment arm. EPAG, eltrombopag.

We analysed the frequencies of patients with mutations in the top 11 genes stratified by baseline MDS risk, defined using the International Prognostic Scoring System (IPSS), as previously reported [5]. Among the cohort of IPSS high‐risk patients, the frequencies of mutations in *TET2* and *BCOR* were higher in the placebo arm, whereas the frequencies of mutations in *NRAS* and *U2AF1* were higher in the eltrombopag arm (Figure S3). Analysis of the frequency of mutations for each arm according to progression status showed more frequent mutations in *TP53* in patients with disease progression in both treatment arms. Additionally, mutations in *TET2* in the placebo arm were less frequent in patients with disease progression. In the eltrombopag arm, mutations in *RUNX1* and *NRAS* were more frequent in patients with progressive disease, whereas mutations in *BCOR*, *DNMT3A*, *SRFS2* and *SF3B1* seemed to be less frequent in patients with disease progression (Figure 1C–F).

In patients with disease progression (*N* = 75; eltrombopag, *n* = 37; placebo, *n* = 38), mutations in *ASXL1*, *NRAS*, *RUNX1* and *TET2* were more frequent in the eltrombopag arm, whereas mutations in *BCOR*, *DNMT3A* and *SRFS2* were more frequent in the placebo arm (Figure 1C,D). In patients without disease progression (*N* = 136; eltrombopag, *n* = 64; placebo, *n* = 72), the frequencies of mutations in the top 11 mutated genes were similar in both arms, except for *TET2*, which was less frequent in the eltrombopag arm (Figure 1E,F). Similar results were observed for patients with or without progression to AML (Figure S4).

Kaplan–Meier analyses of progression‐free survival (PFS) according to the presence or absence of baseline mutations and treatment arm revealed that PFS was shorter in patients with baseline mutations in *TP53*, irrespective of the treatment arm (Figure 2) [10]. Additionally, there was a tendency towards shorter PFS in the eltrombopag arm among patients with versus patients without baseline mutations in *NRAS*. This was not observed in the placebo arm. When comparing the outcome between the two arms according to baseline mutation status, there was a tendency towards worse PFS in the eltrombopag arm among patients carrying baseline mutations in *TP53* (hazard ratio [HR] 1.93), *ASXL1*, *NRAS*, *RUNX1* or *TET2* (all HRs > 2) (Figure 2), but not in other genes (Figure S5). Similar results were obtained for times to progression to AML (Figure S6).

**FIGURE 2:**
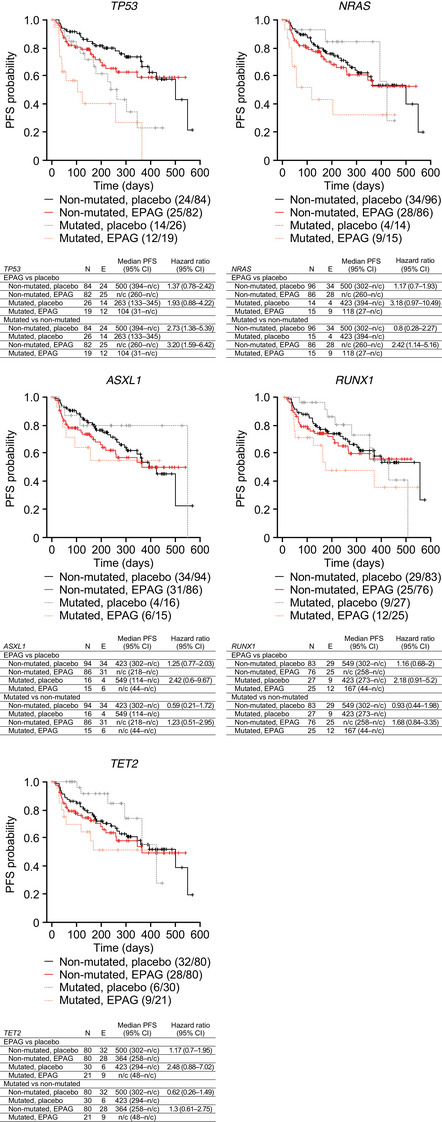
Kaplan–Meier plots of progression‐free survival (PFS) according to treatment and baseline gene mutation status (*TP53*, *NRAS*, *ASXL1*, *RUNX1* and *TET2*) in patients with myelodysplastic syndromes (MDS). Effect of genetic alterations in response to treatment with eltrombopag (EPAG) compared with placebo, both in combination with azacitidine, on the PFS of patients with MDS. See Supporting Information, Figure S5 for Kaplan–Meier plots of PFS for *BCOR*, *DNMT3A*, *SF3B1*, *SRSF2*, *STAG2* and *U2AF1*. The statistical analyses were not adjusted for multiplicity. CI, confidence interval; *E*, number of patients with an event; *N*, number of evaluable patients; n/c, not calculable.

The clinical results from SUPPORT were unexpected, considering that other studies in similar patient populations have demonstrated that eltrombopag monotherapy has a well‐tolerated safety profile and favourable outcomes in patients with MDS with thrombocytopenia [11, 12, 13]. Moreover, there were no signals that were likely to predict the outcomes of SUPPORT in the earlier trial phases of eltrombopag in combination with azacitidine in patients with MDS [3, 4].

NGS revealed a highly variable genetic landscape across both arms, which is consistent with the genetic landscapes reported in other studies [6, 9]. The mutational landscape was broadly similar between the two treatment arms.

Based on the Kaplan–Meier plots of PFS, it seems that *TET2* mutations carried a lower HR than non‐mutant *TET2* (HR 0.62; 95% CI 0.26–1.49) among patients treated with azacitidine alone, but this benefit was lost in the eltrombopag arm (HR 1.3; 95% CI 0.61–2.75). Additionally, the time to disease progression tended to be shorter in patients carrying mutations in *TP53*, *NRAS*, *ASXL1*, *RUNX1* or *TET2* in the eltrombopag arm versus the placebo arm. *TP53*, *RUNX1* and NRAS mutations have been previously reported to be associated with severe thrombocytopenia and elevated blast percentage in patients with MDS [8]. Therefore, it is possible that these patients are less susceptible to stimulation of megakaryopoiesis by eltrombopag, while being more prone to its stimulatory effect on immature blasts.

No statistical testing was performed for any comparisons because the study was not adequately powered for this. Furthermore, this study was limited by sequencing of peripheral blood DNA and the low frequencies of mutations in individual genes, which yielded small subgroups. This, together with the lack of clinical follow‐up, makes it difficult to draw conclusive interpretations, despite interesting trends for some molecular subgroups. Finally, other factors might play a role beyond a direct impact on mutant clones, such as potential antagonistic effects of azacitidine and eltrombopag in regard to normal haematopoiesis.

## AUTHOR CONTRIBUTIONS

Pedro Marques Ramos, Jeea Choi, Catarina D. Campbell, Ying A. Wang and Celine Pallaud conducted the analyses and contributed to the interpretation and reporting of the data. Michael Dickinson, Amit Verma, Moshe Mittelman, Uwe Platzbecker, Honar Cherif and Pierre Fenaux served as investigators in this study, enrolling patients. All authors contributed to data interpretation, reviewed and provided their comments on this manuscript and approved the final version.

## CONFLICT OF INTEREST STATEMENT

Pedro Marques Ramos, Jeea Choi, Catarina D. Campbell, Ying A. Wang and Celine Pallaud are employees of Novartis Pharmaceuticals Corporation. Michael Dickinson has participated in speakers’ bureaus for Novartis Pharmaceuticals Corporation and received research grants from Novartis Pharmaceuticals Corporation and GlaxoSmithKline. Amit Verma has received research funding from GlaxoSmithKline, Celgene and Bristol‐Myers Squibb and has received personal fees from Stelexis Therapeutics. Moshe Mittelman has received research funding from Novartis Pharmaceuticals Corporation and has participated in speakers’ bureaus for Novartis Pharmaceuticals Corporation. Uwe Platzbecker has received honoraria and research funding from Amgen and Novartis Pharmaceuticals Corporation. Honar Cherif has received honoraria and research funding from GlaxoSmithKline (Novartis) and honoraria from Amgen. Pierre Fenaux has received research funding from Celgene, Astex Pharmaceuticals, Jazz Pharmaceuticals and Aprea Therapeutics and has received honoraria from Celgene, Astex Pharmaceuticals and Jazz Pharmaceuticals.

## ETHICS STATEMENT

The study was conducted in accordance with the Declaration of Helsinki and an independent ethics committee or institutional review board for each study site approved the study protocol.

## PATIENT CONSENT STATEMENT

All patients provided written informed consent to participate in the trial (ClinicalTrials.gov: NCT02158936).

## Supporting information

Supporting Information

## Data Availability

The SUPPORT trial data availability is in accordance with the criteria and process described on http://www.clinicalstudydatarequest.com/. A complete list of the SUPPORT study principal investigators can be found in the SUPPORT study publication.
