# Supplementary material for: Next‐generation sequencing of baseline genetic mutations and outcomes of eltrombopag and azacitidine therapy in patients with myelodysplastic syndromes and thrombocytopenia: Data from the SUPPORT clinical trial
Source: EJHaem. 2023 May 22;4(3):876–81. doi: 10.1002/jha2.694 (PMC10435669; doi:10.1002/jha2.694)

# Supplemental Digital Content

## Materials and Methods

## Overview of the SUPPORT trial

The design of the SUPPORT trial is described in more detail elsewhere.^1^

This was a randomized, double-blind, placebo-controlled, international, multicenter, phase 3 study of patients aged ≥18 years (≥20 years in Taiwan) with International Prognostic Scoring System^2^ Int-1, Int-2, or high-risk myelodysplastic syndromes (MDS) and at least 1 platelet count of <75 × 10^9^/L within 28 days prior to the first azacitidine dose. Eligible patients were randomized 1:1 to eltrombopag or placebo, both in combination with azacitidine. Patients received at least 6 cycles of azacitidine with eltrombopag or placebo administered from day 1, which was to be continued for as long as benefit was derived or until disease progression, unacceptable toxicity, or death.

Disease progression was recorded according to investigator’s judgment based on the modified 2006 International Working Group criteria for MDS disease response and progression,^3^ and according to central review based on bone marrow and/or peripheral blast assessment.

The study was conducted in accordance with the Declaration of Helsinki and an independent ethics committee or institutional review board for each study site approved the study protocol. All patients provided written informed consent to participate in the trial (ClinicalTrials.gov: NCT02158936).

## Next-generation sequencing

A total of 329 samples were available for next-generation sequencing (NGS). Of these, 12 were excluded from the analyses owing to sequencing issues or unsolved collection quality control issues. Therefore, NGS data are presented for 211 samples collected at baseline (n=101 eltrombopag; n=110 placebo).

Samples of whole blood were collected in PAXgene tubes at baseline from consenting patients. Genomic DNA was extracted using Maxwell^®^ whole blood DNA extraction kits (Promega, Madison, WI). The extracted DNA was sheared using an ultrasonicator (Covaris, Woburn, MA) followed by end repair, A-tailing, indexed adaptor ligation, and polymerase chain reaction amplification using the TruSeq Nano Library Preparation kit (Illumina, San Diego, CA). Using this kit, the constructed libraries were captured with baits targeting the coding sequences of 356 genes and the introns involved in 45 reported gene fusions. The captured libraries were sequenced to a targeted depth of coverage of 1000× by paired-end sequencing.

The NGS panel used here targeted 365 genes involved in hematopoietic malignancies. This report focuses on 53 genes previously reported to be mutated in MDS,^4-7^ including a subset of 18 genes associated with poor prognosis (Supplemental Digital Content, Table SIII).^8,9^

Sequencing reads were aligned to the human reference genome (build hg19) using a Burrows-Wheeler Aligner.^10^ The aligned reads were then cleaned with Picard to mark polymerase chain reaction duplicates and record the percentage of duplicated reads.^11^ Then, the Genome Analysis ToolKit was used for local realignment and base quality score recalibration.^12,13^ Single-nucleotide variants (SNVs) were identified with MuTect.^14^ Short insertion/deletion (indels) events were called using Pindel.^15^ Structural variants were identified based on deviations in the depth of coverage and with PureCN.^16^ Chromosomal rearrangements were identified using Socrates.^17^

Potential germline SNVs and indels were flagged based on being present in the Exome Sequencing Project database^18^ or the Exome Aggregation Consortium,^19^ and being absent in the Catalog of Somatic Mutations in Cancer.^20^ Potential artefactual SNVs and indels were flagged based on the low base quality of the variant allele, regions of low mapping quality, and repetitive regions. Flagged variants were not considered in the downstream analysis. Copy number variations were considered amplifications if the estimated copy number was greater than 6 and considered homozygous deletions if the estimated copy number was less than 0.5. Translocations were removed from downstream analysis if 1 breakpoint was not in a gene, both breakpoints were in the same gene, or neither gene was a targeted fusion partner.

## Data analysis

The frequencies of patients carrying acute myeloid leukemia (AML)/MDS-associated mutations at baseline were compared between treatment arms using contingency tables and applying 2-sided Fisher’s exact test. A confidence interval (CI) was calculated for the odds ratio (OR) using the (central) Fisher’s exact test. Cox proportional hazards models were used to examine which biomarkers were associated with progression-free survival (PFS) or time to progression to AML. Subgroup analyses were done to compare the clinical outcome between the 2 treatment arms within each mutation status group (mutant vs non-mutant) and to compare clinical outcomes between the mutation status groups within each treatment arm. The Kaplan-Meier method was also used to plot PFS (defined as the time from randomization to either disease progression or death) and time to progression to AML for each biomarker and treatment arm. Patients who were event-free at the time of analysis were censored at the time of last contact. As this study was not powered to assess specific biomarker-related hypotheses, the statistical analyses of the data are considered exclusively exploratory in nature. No adjustment for multiple comparisons was conducted and no statistical testing was performed for any comparisons. Clonal evolution was examined by plotting the changes in variant allelic frequencies over time in individual patients from whom NGS data were available at baseline and at least one follow-up time point. All data analyses were performed using the R programming language.

**Supplemental references**

1. Dickinson M, Cherif H, Fenaux P, et al. Azacitidine with or without eltrombopag for first-line treatment of intermediate- or high-risk MDS with thrombocytopenia. *Blood*. 2018;132(25):2629-2638.

2. Greenberg P, Cox C, LeBeau MM, et al. International scoring system for evaluating prognosis in myelodysplastic syndromes. *Blood*. 1997;89:2079-2088.

3. Cheson BD, Greenberg PL, Bennett JM, et al. Clinical application and proposal for modification of the International Working Group (IWG) response criteria in myelodysplasia. *Blood*. 2006;108(2):419-425.

4. Haferlach T, Nagata Y, Grossmann V, et al. Landscape of genetic lesions in 944 patients with myelodysplastic syndromes. *Leukemia*. 2014;28(2):241-247.

5. Ley TJ, Miller C, Ding L, et al. Genomic and epigenomic landscapes of adult de novo acute myeloid leukemia. *N Engl J Med*. 2013;368(22):2059-2074.

6. Papaemmanuil E, Gerstung M, Bullinger L, et al. Genomic classification and prognosis in acute myeloid leukemia. *N Engl J Med*. 2016;374(23):2209-2221.

7. Papaemmanuil E, Gerstung M, Malcovati L, et al. Clinical and biological implications of driver mutations in myelodysplastic syndromes. *Blood*. 2013;122(22):3616-3627.

8. Makishima H, Yoshizato T, Yoshida K, et al. Dynamics of clonal evolution in myelodysplastic syndromes. *Nat Genet*. 2017;49(2):204-212.

9. Xu F, Wu L-Y, He Q, et al. Exploration of the role of gene mutations in myelodysplastic syndromes through a sequencing design involving a small number of target genes. *Sci Rep*. 2017;7:43113.

10. Fenaux P, Mufti GJ, Hellstrom-Lindberg E, et al. Efficacy of azacitidine compared with that of conventional care regimens in the treatment of higher-risk myelodysplastic syndromes: a randomised, open-label, phase III study. *Lancet Oncol*. 2009;10(3):223-232.

11. Broad Institute. Picard tools. 2018. <http://broadinstitute.github.io/picard/>. Accessed February 23, 2022.

12. DePristo MA, Banks E, Poplin R, et al. A framework for variation discovery and genotyping using next-generation DNA sequencing data. *Nature Genetics*. 2011;43:491.

13. McKenna A, Hanna M, Banks E, et al. The Genome Analysis Toolkit: a MapReduce framework for analyzing next-generation DNA sequencing data. *Genome Res*. 2010;20(9):1297-1303.

14. Cibulskis K, Lawrence MS, Carter SL, et al. Sensitive detection of somatic point mutations in impure and heterogeneous cancer samples. *Nat Biotechnol*. 2013;31(3):213-219.

15. Ye K, Schulz MH, Long Q, Apweiler R, Ning Z. Pindel: a pattern growth approach to detect break points of large deletions and medium sized insertions from paired-end short reads. *Bioinformatics*. 2009;25(21):2865-2871.

16. Riester M, Singh AP, Brannon AR, et al. PureCN: copy number calling and SNV classification using targeted short read sequencing. *Source Code Biol Med*. 2016;11:13.

17. Schroder J, Hsu A, Boyle SE, et al. Socrates: identification of genomic rearrangements in tumour genomes by re-aligning soft clipped reads. *Bioinformatics*. 2014;30(8):1064-1072.

18. NHLBI Exome Sequencing Project (ESP). Exome variant server. 2018. <http://evs.gs.washington.edu/EVS/>. Accessed February 23, 2022.

19. Lek M, Karczewski KJ, Minikel EV, et al. Analysis of protein-coding genetic variation in 60,706 humans. *Nature*. 2016;536(7616):285-291.

20. Wellcome Sanger Institute. COSMIC: Catalogue of somatic mutations in cancer. 2018. <https://cancer.sanger.ac.uk/cosmic>. Accessed February 23, 2022.

**Supplemental Digital Content, Table SI. Genes evaluated by next-generation sequencing.**

| **Genes that are frequently mutated in MDS or AML** | | | | |  | | **Genes associated with disease progression or poor prognosis** | | | |
| --- | --- | --- | --- | --- | --- | --- | --- | --- | --- | --- |
| **Gene** | **Reference** | **Gene** | | **Reference** |  | | | **Gene** | | **Reference** |
| *ASXL1* | 2–4 | *MLL2* | | 2,3 |  | | | *ASXL1* | | 5 |
| *ATRX* | 2,3 | *MLL3* | | 2 |  | | | *DNMT3A* | | 6 |
| *BCOR* | 2–4 | *MPL* | | 2–4 |  | | | *EZH2* | | 6 |
| *BRAF* | 2,3 | *MYC* | | 2 |  | | | *FLT3* | | 5 |
| *CBL* | 2–4 | *NF1* | | 2–4 |  | | | *GATA2* | | 5 |
| *CBLB* | 2 | *NPM1* | | 1–4 |  | | | *IDH1* | | 5,6 |
| *CDKN2A* | 2,3 | *NRAS* | | 1–4 |  | | | *IDH2* | | 5,6 |
| *CEBPA* | 1–4 | *PHF6* | | 1–4 |  | | | *KRAS* | | 5 |
| *CREBBP* | 2,3 | *PTEN* | | 2,3 |  | | | *NPM1* | | 5 |
| *CUX1* | 2,3 | *PTPN11* | | 1–4 |  | | | *NRAS* | | 5 |
| *DNMT3A* | 1–4 | *RAD21* | | 1–4 |  | | | *PTPN11* | | 5 |
| *EP300* | 2,3 | *RB1* | | 2 |  | | | *RUNX1* | | 5,6 |
| *ETV6* | 2–4 | *RUNX1* | | 1–4 |  | | | *SRSF2* | | 6 |
| *EZH2* | 1–4 | *SF1* | | 2,4 |  | | | *STAG2* | | 5,6 |
| *FBXW7* | 2,4 | *SF3B1* | | 2–4 |  | | | *TET2* | | 5 |
| *FLT3* | 1–4 | *SH2B3* | | 2,3 |  | | | *TP53* | | 5,6 |
| *GATA2* | 2–4 | *SMC1A* | | 1,4 |  | | | *WT1* | | 5,6 |
| *GNAS* | 2–4 | *SMC3* | | 1,4 |  | | | *ZRSR2* | | 5 |
| *IDH1* | 1–4 | *SRSF2* | | 2–4 |  | | |  | |  |
| *IDH2* | 1–4 | *STAG2* | | 1–4 |  | | |  | |  |
| *IKZF1* | 2 | *TET2* | | 1–4 |  | | |  | |  |
| *JAK2* | 2–4 | *TP53* | | 1–4 |  | | |  | |  |
| *KDM5A* | 2 | *U2AF1* | | 1–4 |  | | |  | |  |
| *KDM6A* | 2,3 | *U2AF2* | | 2,4 |  | | |  | |  |
| *KIT* | 1–4 | *WT1* | | 1–3 |  | | |  | |  |
| *KMT2A* | 2 | *ZRSR2* | | 2–4 |  | | |  | |  |
| *KRAS* | 1–4 |  |  | |  |  | | |  | |

AML=acute myeloid leukemia; MDS=myelodysplastic syndromes.

1. Ley TJ, Miller C, Ding L *et al*. Genomic and epigenomic landscapes of adult *de novo* acute myeloid leukemia. *N Engl J Med* 2013;368:2059–2074.

2. Papaemmanuil E, Gerstung M, Bullinger L *et al*. Genomic classification and prognosis in acute myeloid leukemia. *N Engl J Med* 2016;374:2209–2221.

3. Papaemmanuil E, Gerstung M, Malcovati L *et al*. Clinical and biological implications of driver mutations in myelodysplastic syndromes. *Blood* 2013;122:3616–3627.

4. Haferlach T, Nagata Y, Grossmann V *et al*. Landscape of genetic lesions in 944 patients with myelodysplastic syndromes. *Leukemia* 2014;28:241–247.

5. Makishima H, Yoshizato T, Yoshida K *et al*. Dynamics of clonal evolution in myelodysplastic syndromes. *Nat Genet* 2017;49:204–212.

6. Xu F, Wu LY, He Q *et al*. Exploration of the role of gene mutations in myelodysplastic syndromes through a sequencing design involving a small number of target genes. *Sci Rep* 2017;7:43113.

**Supplemental Digital Content, Table SII.** **Patient** **demographics and baseline characteristics.**

| **Demographic variable** | **NGS population** | | **NGS population**  **N=211** | **ITT  population**  **N=356** |
| --- | --- | --- | --- | --- |
|  | **EPAG**  **N=101** | **Placebo**  **N=110** |  |  |
| **Age (years)** |  |  |  |  |
| Mean ± SD | 66.9 ± 12.94 | 69.7 ± 10.28 | 68.4 ± 11.68 | 68.8 ± 11.76 |
| Median (range) | 69.0 (24-89) | 70.0 (32-88) | 69.0 (24-89) | 70.0 (24-89) |
| **Age category (years), n (%)** |  |  |  |  |
| ≥18-64 | 31 (31) | 33 (30) | 64 (30) | 100 (28) |
| ≥65-74 | 43 (43) | 40 (36) | 83 (39) | 132 (37) |
| ≥75-84 | 22 (22) | 29 (26) | 51 (24) | 103 (29) |
| ≥85 | 5 (5) | 8 (7) | 13 (6) | 21 (6) |
| **Sex, n (%)** |  |  |  |  |
| Male | 62 (61) | 72 (65) | 134 (64) | 234 (66) |
| Female | 39 (39) | 38 (35) | 77 (36) | 122 (34) |
| **Race, n (%)** |  |  |  |  |
| White | 89 (88) | 100 (91) | 189 (90) | 294 (83) |
| East Asian/Japanese/  Southeast Asian | 9 (9) | 8 (7) | 17 (8) | 49 (14) |
| Other | 1 (1) | 2 (2) | 3 (1) | 8 (2) |
| Missing | 2 (2) | 0 | 2 (<1) | 3 (<1) |
| **IPSS risk score, n (%)** |  |  |  |  |
| Int-1 | 39 (39) | 40 (36) | 79 (37) | 125 (35) |
| Int-2 | 44 (44) | 52 (47) | 96 (45) | 160 (45) |
| HR | 18 (18) | 18 (16) | 36 (17) | 71 (20) |
| **Bone marrow blast count (local data), n (%)** |  |  |  |  |
| <5% | 37 (37) | 30 (27) | 67 (32) | 109 (31) |
| 5-10% | 26 (26) | 41 (37) | 67 (32) | 109 (31) |
| 11-20% | 33 (33) | 33 (30) | 66 (31) | 115 (32) |
| 21-30%^a^ | 5 (5) | 6 (5) | 11 (5) | 21 (6) |
| **Platelet count, n (%)** |  |  |  |  |
| <10 × 10^9^/L | 4 (4) | 7 (6) | 11 (5) | 20 (6) |
| ≥10 to <20 × 10^9^/L | 19 (19) | 19 (17) | 38 (18) | 65 (18) |
| ≥20 to <50 × 10^9^/L | 50 (50) | 50 (45) | 100 (47) | 167 (47) |
| ≥50 to <100 × 10^9^/L | 27 (27) | 34 (31) | 61 (29) | 102 (29) |
| **Platelet transfusion dependence, n (%)** |  |  |  |  |
| Yes | 14 (14) | 21 (19) | 35 (17) | 66 (19) |
| No | 87 (86) | 89 (81) | 176 (83) | 290 (81) |
| **Karyotype^b^** |  |  |  |  |
| 0 Good | 52 (51) | 53 (48) | 105 (50) | 167 (47) |
| 0.5 Intermediate | 20 (20) | 24 (22) | 44 (21) | 78 (22) |
| 1 Poor | 29 (29) | 33 (30) | 62 (29) | 111 (31) |

^a^Patients with AML by WHO or FAB criteria; ^b^0 Good = normal karyotype, Y alone, del(5q) alone, or del(20q) alone; 0.5 Intermediate = other abnormalities; 1 Poor = abnormalities involving chromosome 7 or those with a complex karyotype (≥3 unassociated abnormalities).
AML=acute myeloid leukemia; EPAG=eltrombopag; FAB=French–American–British; HR=high-risk; Int-1=intermediate-1; Int-2=intermediate-2; IPSS=International Prognostic Scoring System; ITT=intent-to-treat; NGS=next-generation sequencing; SD=standard deviation; WHO=World Health Organization.

**Supplemental Digital Content, Table SIII.** **Distribution of mutations in 53 MDS genes and 18 prognostic genes at baseline.**

| **Number of mutations per patient** | **EPAG**  **N=101** | **Placebo**  **N=110** | **Total**  **N=211** |
| --- | --- | --- | --- |
| **In 53 MDS-related genes, n (%)** |  |  |  |
| 0 | 15 (14.9) | 9 (8.2) | 24 (11.4) |
| 1 | 20 (19.8) | 23 (20.9) | 43 (20.4) |
| 2 | 21 (20.8) | 29 (26.4) | 50 (23.7) |
| 3 | 14 (13.9) | 20 (18.2) | 34 (16.1) |
| 4 | 12 (11.9) | 11 (10.0) | 23 (10.9) |
| ≥5 | 19 (18.8) | 18 (16.4) | 37 (17.5) |
| **In 18 prognostic genes, n (%)** |  |  |  |
| 0 | 24 (23.8) | 14 (12.7) | 38 (18.0) |
| 1 | 24 (23.8) | 37 (33.6) | 61 (28.9) |
| 2 | 23 (22.8) | 29 (26.4) | 52 (24.6) |
| 3 | 8 (7.9) | 10 (9.1) | 18 (8.5) |
| 4 | 11 (10.9) | 10 (9.1) | 21 (10.0) |
| ≥5 | 11 (10.9) | 10 (9.1) | 21 (10.0) |

EPAG=eltrombopag; MDS=myelodysplastic syndromes.

**Supplemental Digital Content, Fig S1. Patient disposition/flow chart.** ^a^Two patients were randomized to EPAG but were not treated; ^b^Patients with mutations in MDS-related genes detected in samples obtained at baseline and/or follow-up.
AML=acute myeloid leukemia; MDS=myelodysplastic syndromes; NGS=next-generation sequencing.


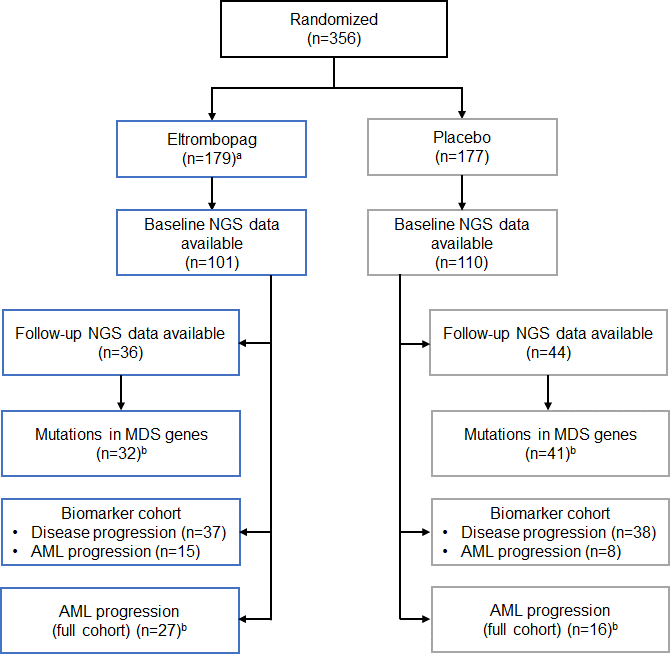


**Supplemental Digital Content, Fig S2. Baseline allelic frequencies in patients with myelodysplastic syndromes treated with eltrombopag or placebo, both in combination with azacitidine.** Box plots show the median (middle line) and the interquartile range (top/bottom) of the allelic frequencies of 11 genes according to treatment arm. The ×s denote the frequency of variants/patients with mutations in each gene. The number of variants/patients is given below the figure. Genes are listed by descending order of allelic frequency in the eltrombopag group. EPAG=eltrombopag.


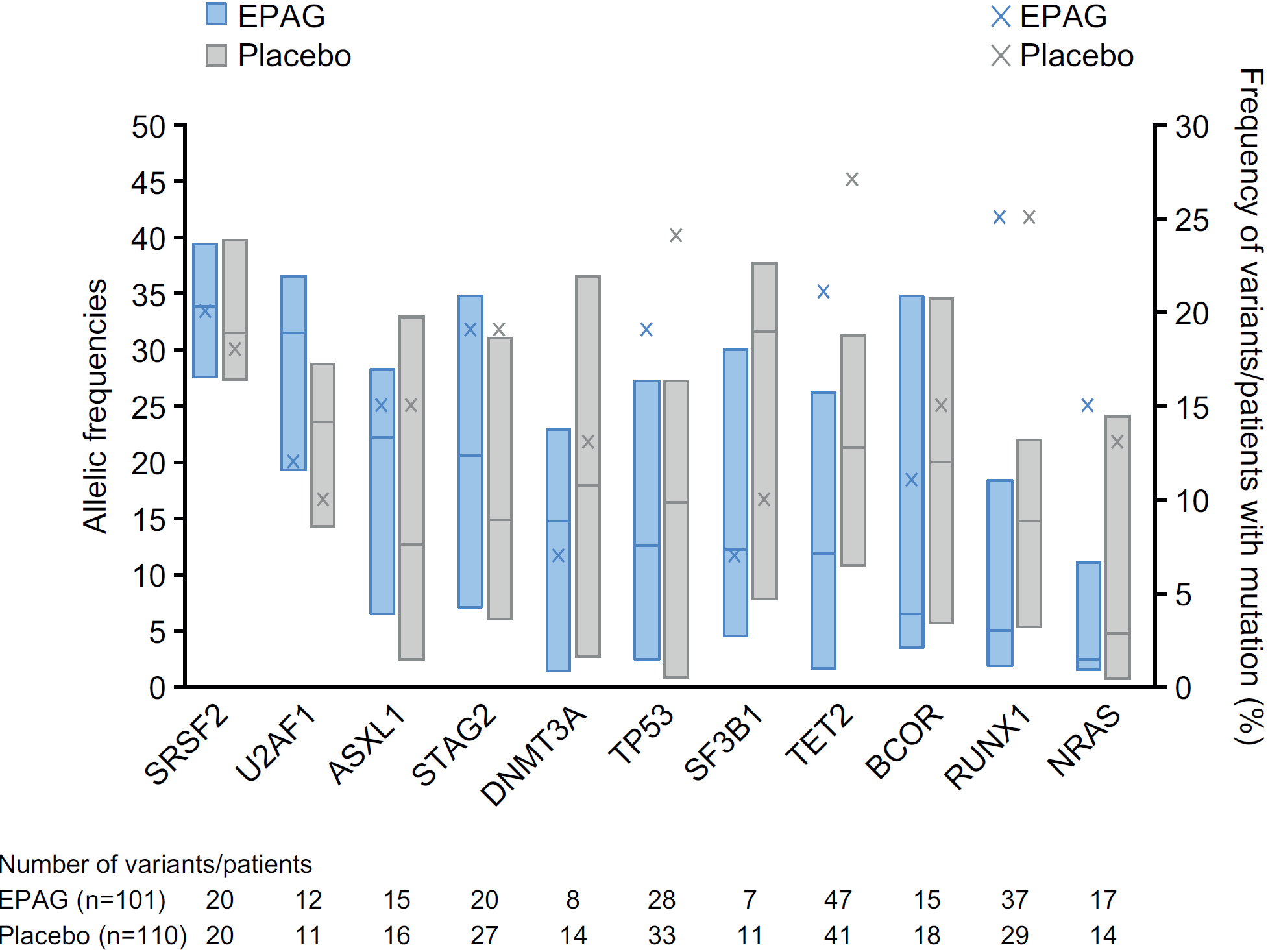


**Supplemental Digital Content, Fig S3. OncoPrints of gene mutations in myelodysplastic syndromes and prognostic genes according to treatment arm and IPSS risk category.** Genomic alterations stratified by baseline risk occurring in the 11 most common genes at baseline in patients with myelodysplastic syndromes. EPAG=eltrombopag; HR=high-risk; Int-1=intermediate-1; Int-2=intermediate-2; IPSS=International Prognostic Scoring System.


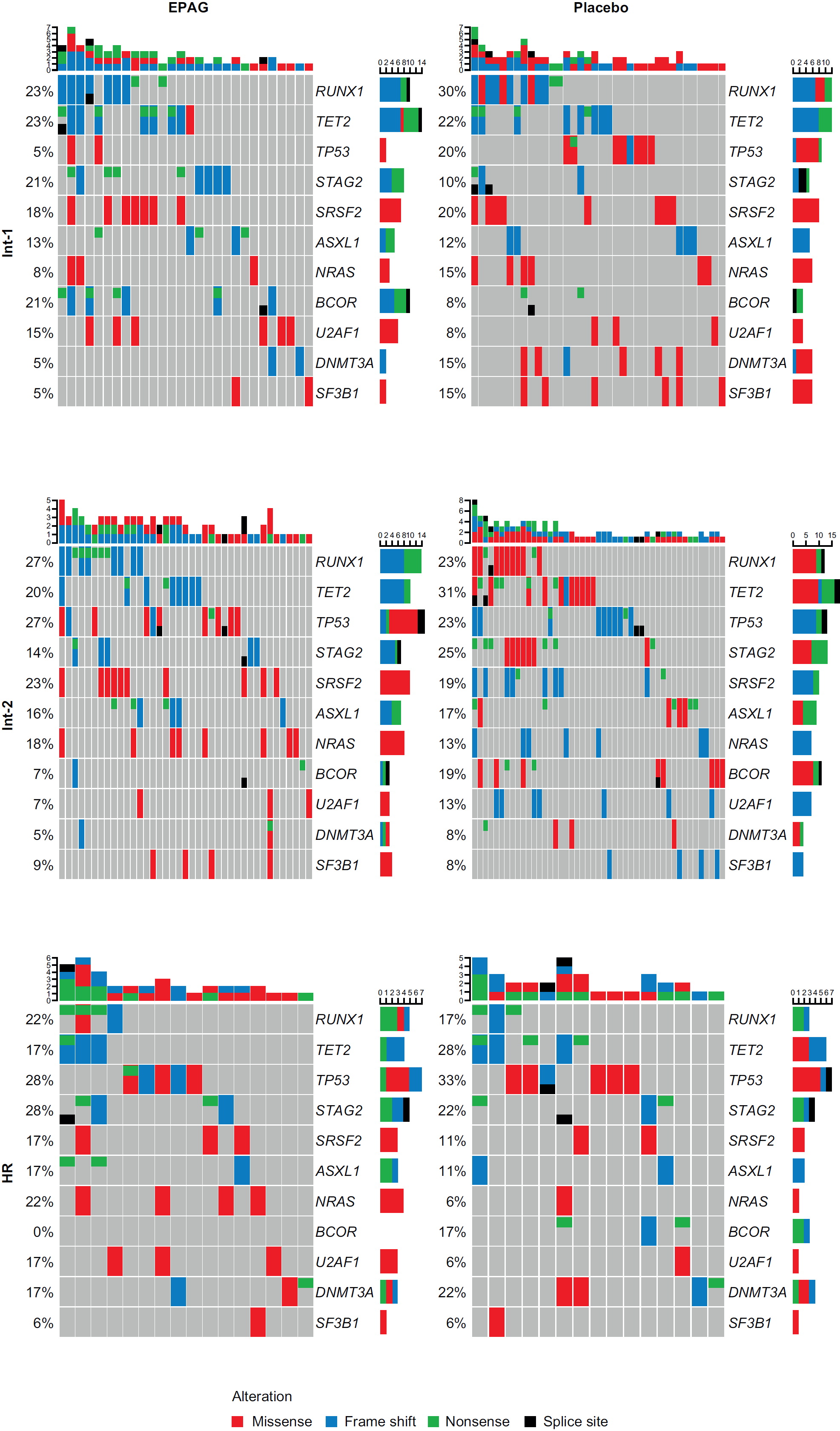


**Supplemental Digital Content, Fig S4. Baseline frequency of gene mutations among patients with or without AML progression.** AML=acute myeloid leukemia; EPAG=eltrombopag.


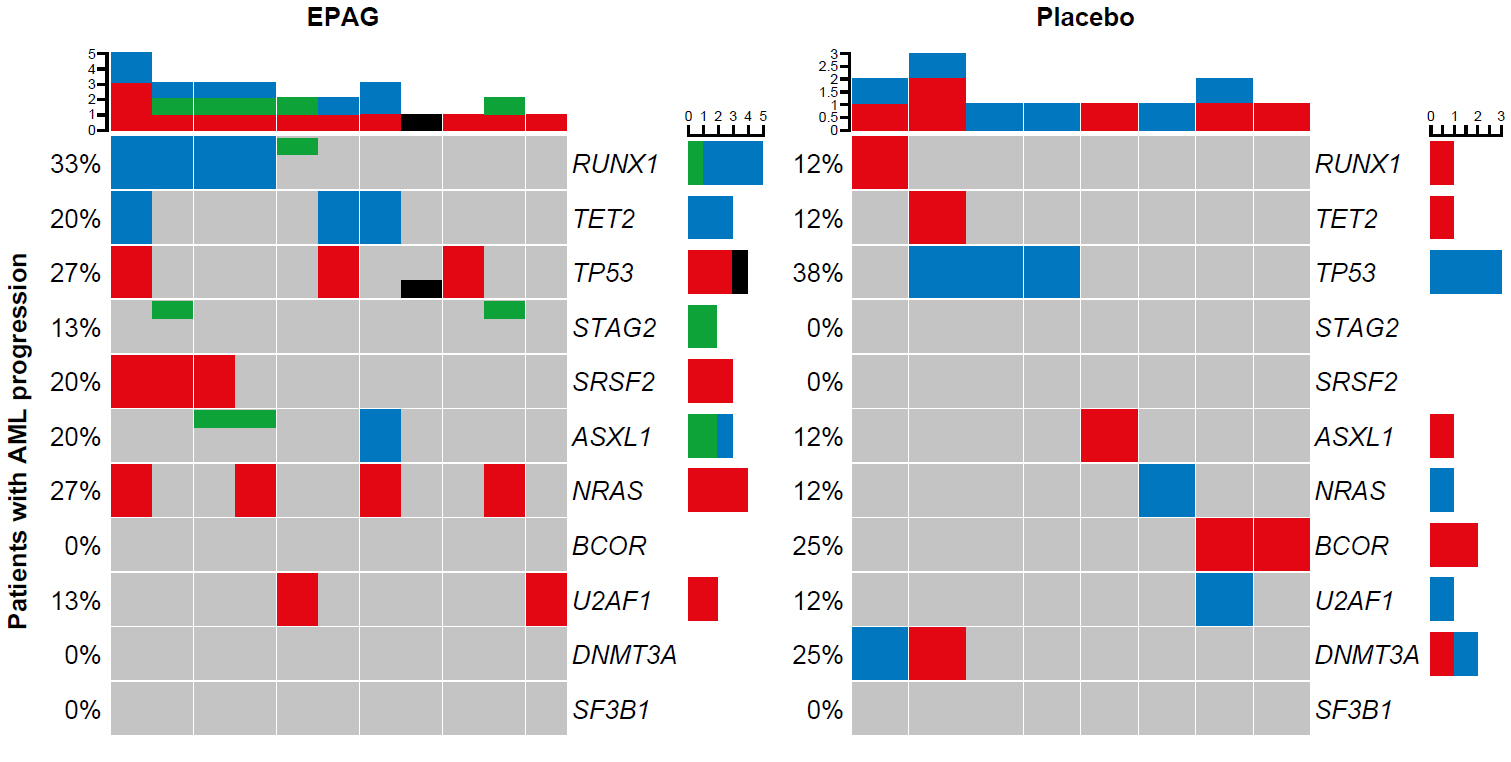


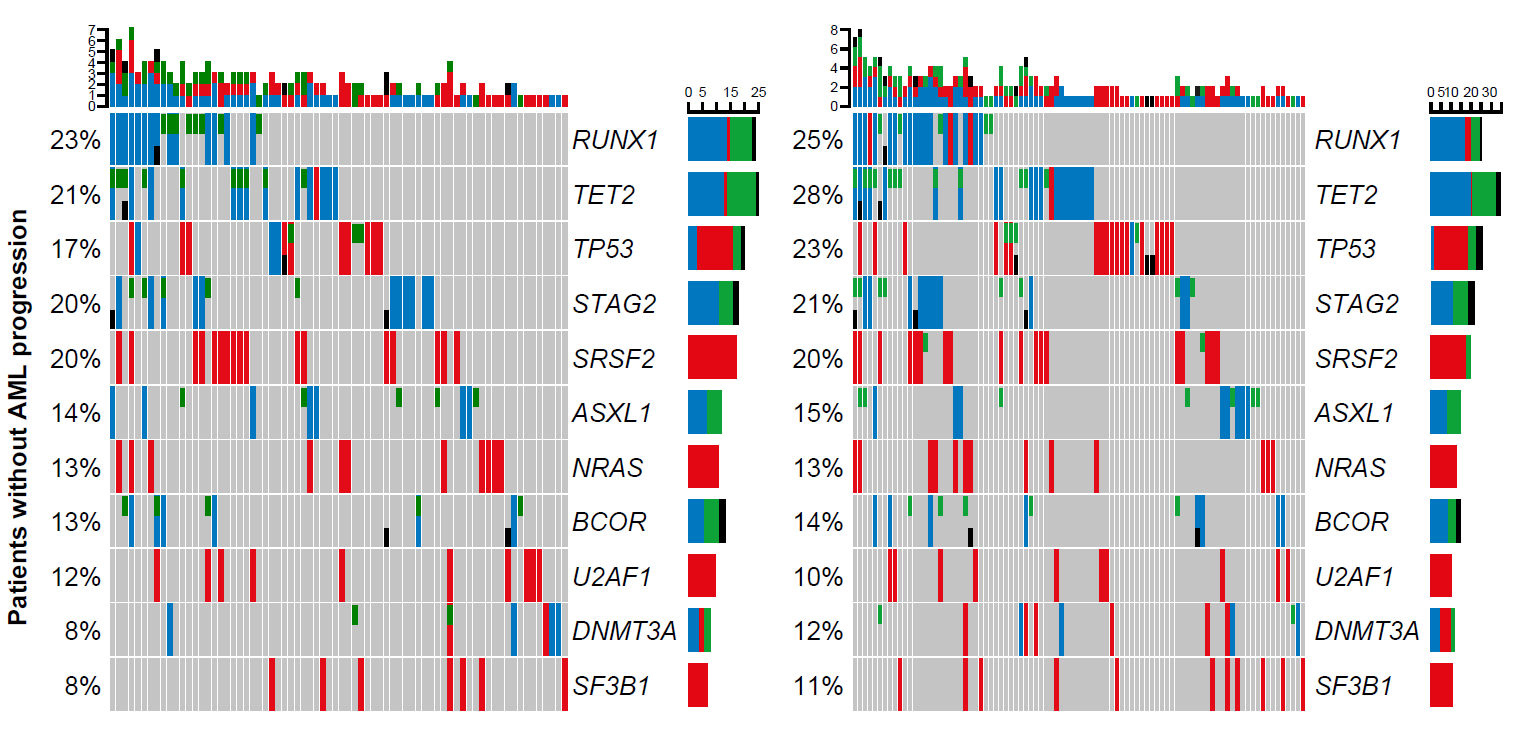


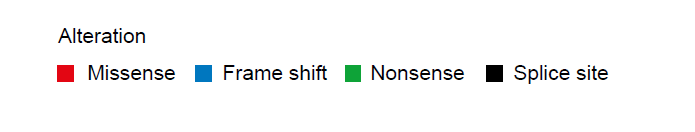


**Supplemental Digital Content, Fig S5. Kaplan-Meier plots of PFS according to treatment received and mutation status at baseline for the genes *BCOR*, *DNMT3A*, *SF3B1*, *SRSF2*, *STAG2*, and *U2AF1.*** The statistical analyses were not adjusted for multiplicity.

CI=confidence interval; E=number of patients with an event; EPAG=eltrombopag; N=number of evaluable patients; n/c=not calculable; PFS=progression-free survival.


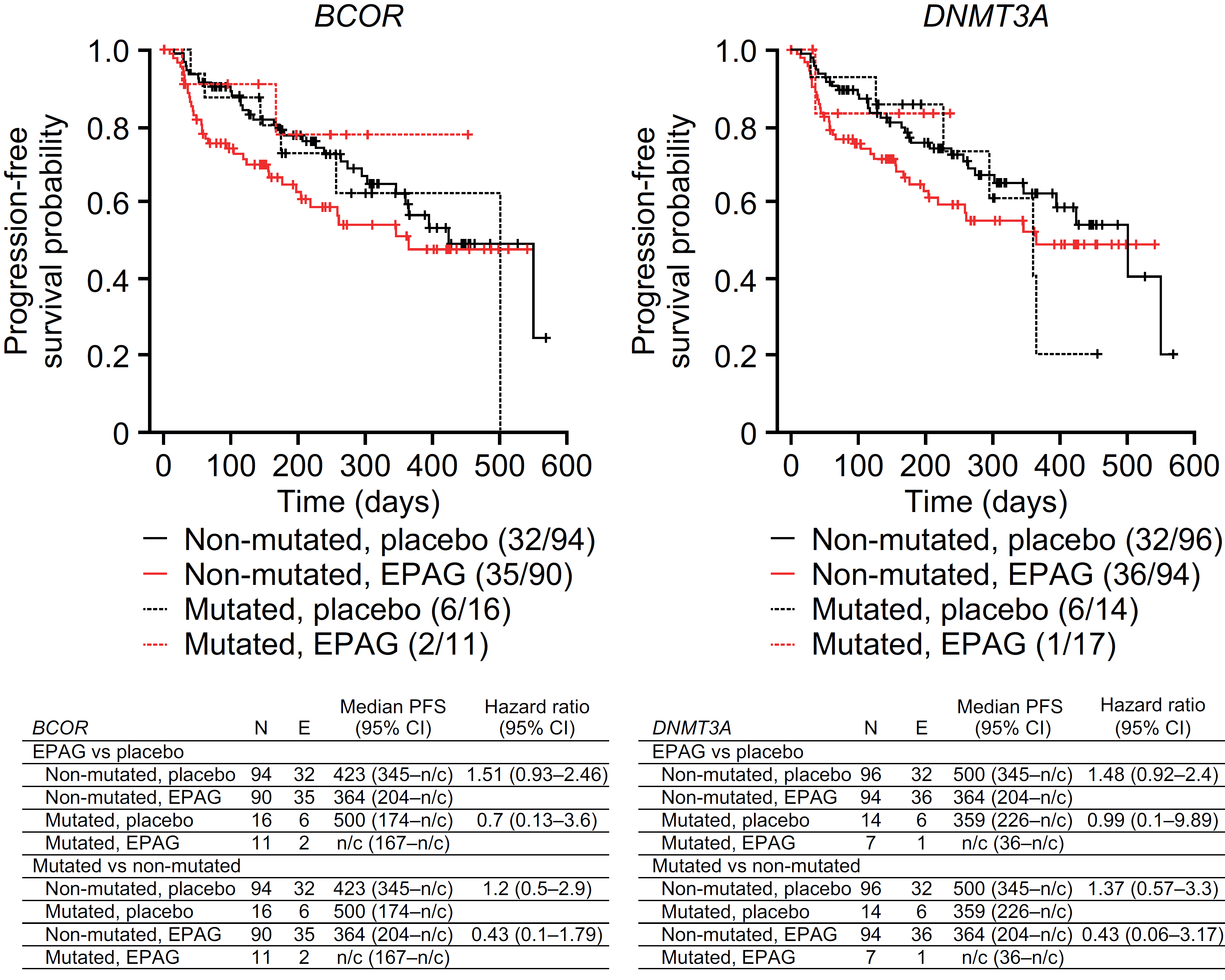


**Fig S5 (continued)**


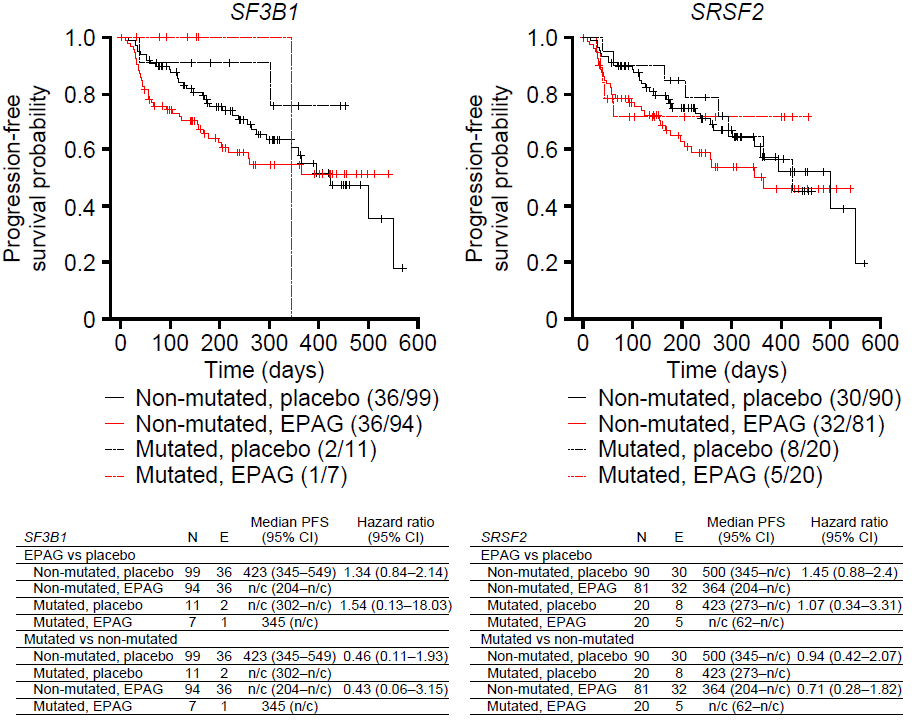


**Fig S5 (continued)**


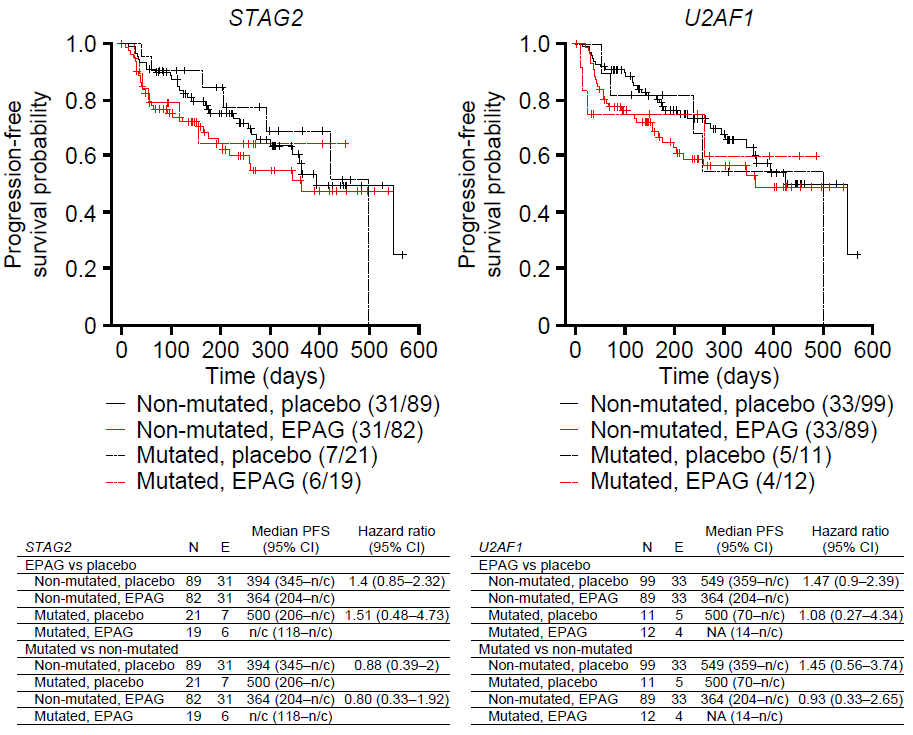


**Supplemental Digital Content, Fig S6. Kaplan-Meier plots of time to AML progression according to treatment received and mutation status at baseline.** Panel A: *TP53*, *NRAS*, *ASXL1*, *RUNX1*, *and TET2.* Panel B: *BCOR*, *DNMT3A*, *SF3B1*, *SRSF2*, *STAG2*, and *U2AF1*.

The statistical analyses were not adjusted for multiplicity. AML=acute myeloid leukemia; CI=confidence interval; EPAG=eltrombopag; E=number of patients with an event; inf= infinity; N=number of evaluable patients; n/c=not calculable.

**Panel A**


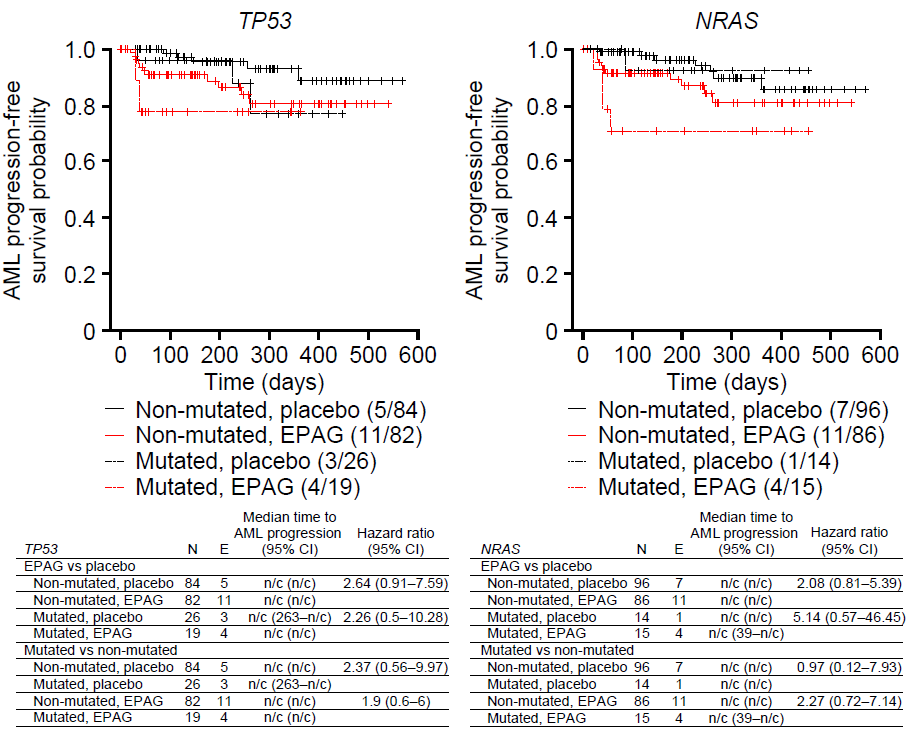


**Panel A (continued)**


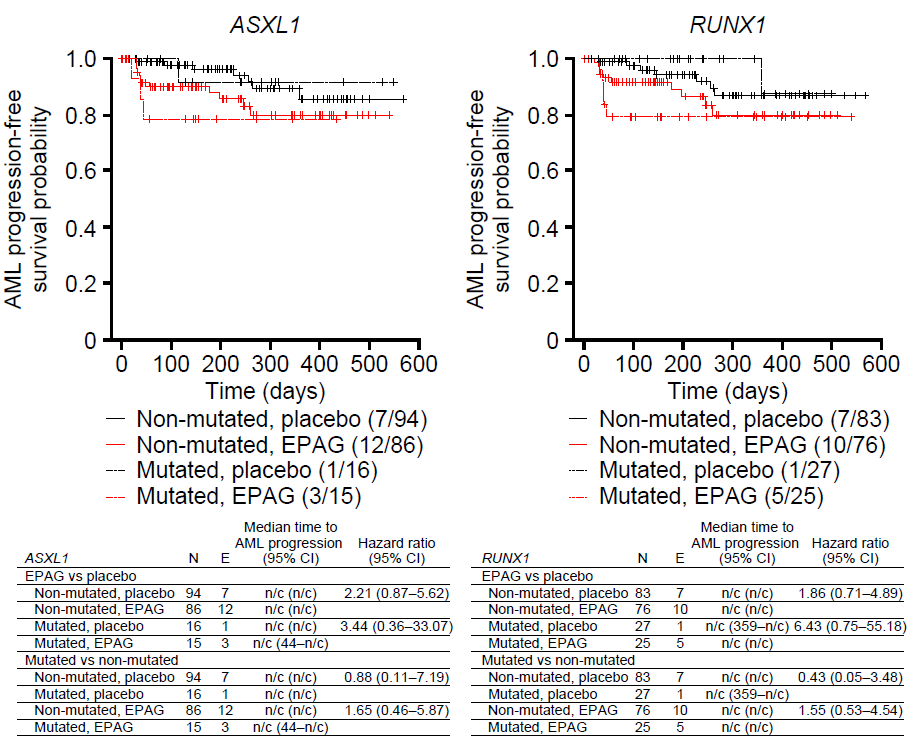


**Panel A (continued)**


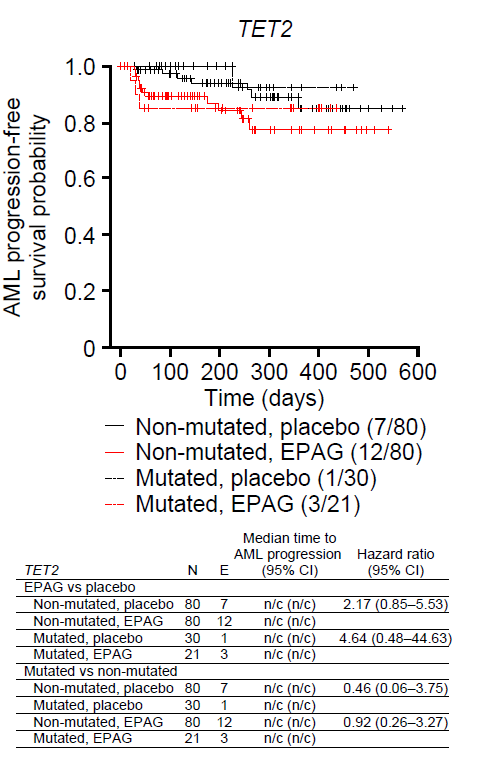


**Panel B**


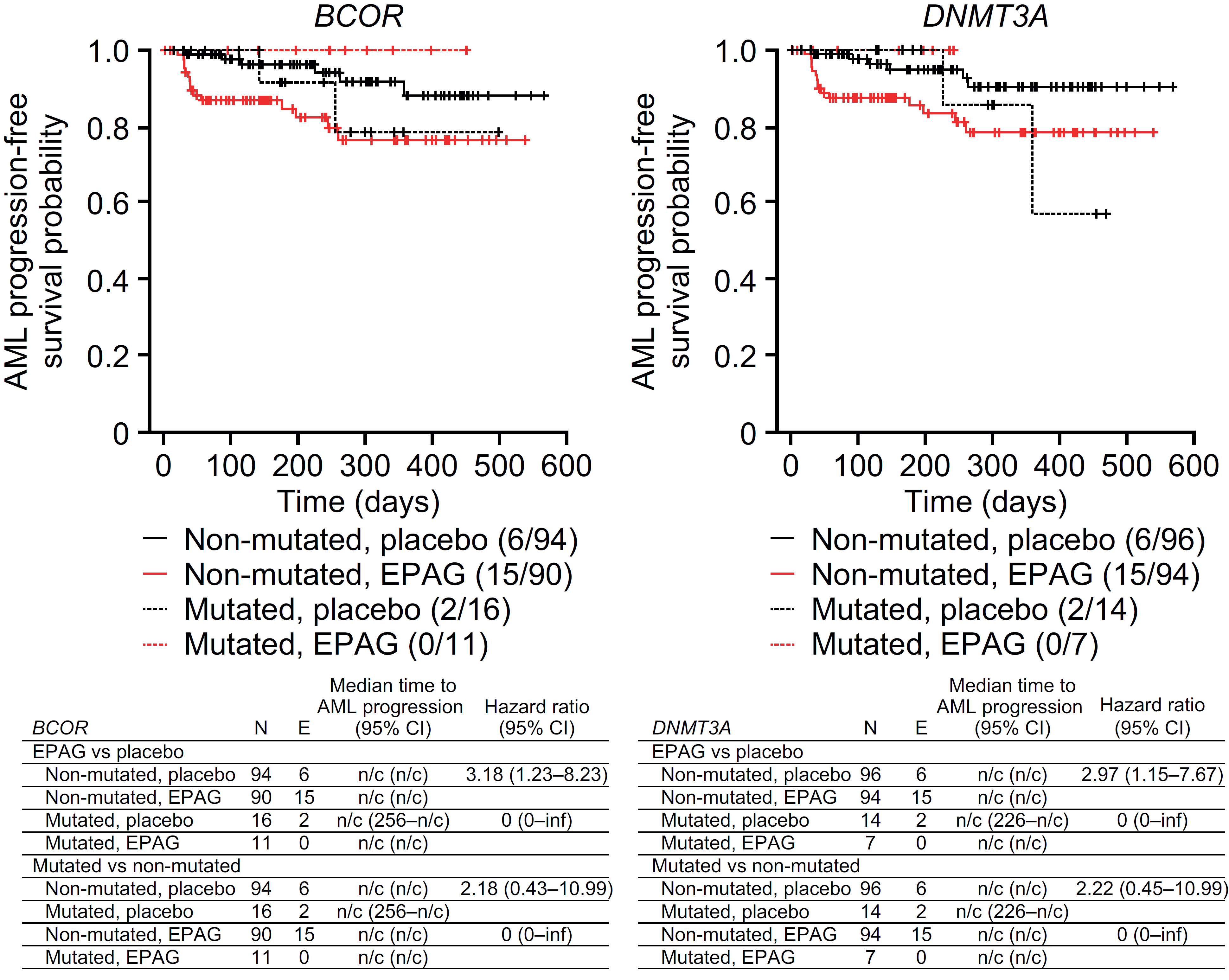


**Panel B (continued)**


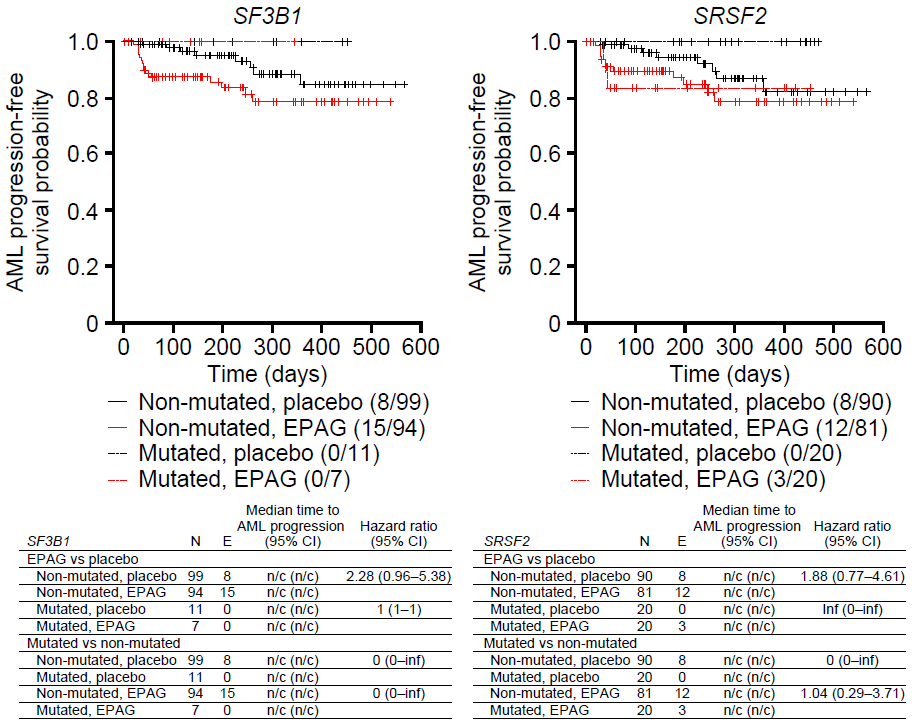


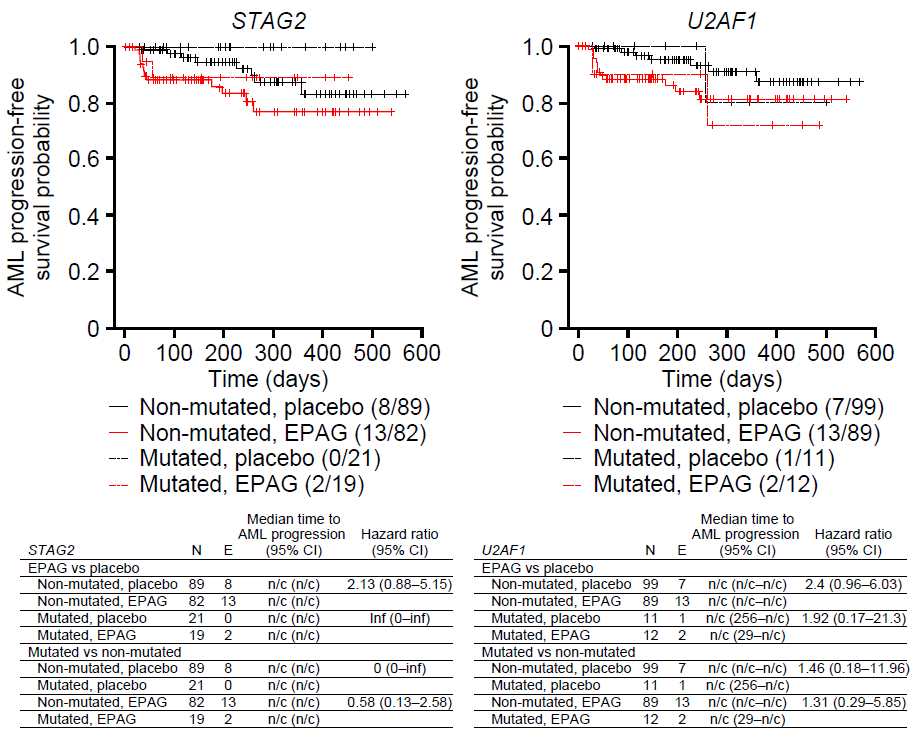

Supplement: Supplementary file 1 — Supporting Information [file JHA2-4-876-s001.docx]
